# Psychometric properties of the Norwegian version of the Patient Health Questionnaire-9 (PHQ-9) in a large female sample of adults with and without eating disorders

**DOI:** 10.1186/s12888-020-03013-0

**Published:** 2021-01-05

**Authors:** Line Wisting, Sverre Urnes Johnson, Cynthia M. Bulik, Ole A. Andreassen, Øyvind Rø, Lasse Bang

**Affiliations:** 1 Division of Mental Health and Addiction, Regional Department for Eating Disorders, Oslo University Hospital, P.O. Box 4956 Nydalen, 0424 Oslo, Norway; 2 Department of Psychology, University of Oslo, Oslo, Norway; 3 Modum Bad Psychiatric Center, Vikersund, Norway; 4 Department of Medical Epidemiology and Biostatistics, Karolinska Institutet, Stockholm, Sweden; 5 Department of Psychiatry, University of North Carolina at Chapel Hill, Chapel Hill, NC USA; 6 Department of Nutrition, University of North Carolina at Chapel Hill, Chapel Hill, NC USA; 7 NORMENT, KG Jebsen Centre for Psychosis Research, Institute of Clinical Medicine, University of Oslo and Division of Mental Health and Addiction, Oslo University Hospital, 0407 Oslo, Norway; 8 Faculty of Medicine, Mental Health and Addiction, University of Oslo, Oslo, Norway

**Keywords:** Depression, Eating disorders, Assessment, Psychology

## Abstract

**Background:**

Internationally, the Patient Health Questionnaire-9 (PHQ-9) is commonly used to assess the frequency and severity of depressive symptoms. However, psychometric properties of the Norwegian version of the PHQ-9 have only been assessed in adolescents. We present normative data for women and an evaluation of the psychometric properties (internal consistency, convergent validity, and factor structure) of the Norwegian PHQ-9 among women with and without eating disorders (ED).

**Methods:**

In this case-control study, a total of 793 females aged 18–78 years (mean 30.39; SD 9.83) completed an online self-report assessment. Measures included the ED100K and Eating Disorder Examination Questionnaire (EDE-Q) to assess ED psychopathology, and the Generalized Anxiety Disorder (GAD) scale and Difficulties in Emotion Regulation Scale Short Form (DERS-SF) to assess symptoms of anxiety and emotion regulation deficits. Participants were categorized into three groups, i.e., previous ED (19.7%, *n* = 148), current ED (36.3%, *n* = 272), and no history of ED (44.0%, *n* = 330), based on self-reported scores on the ED 100 K and the EDE-Q.

**Results:**

Mean PHQ-9 total score for those with a previous history of ED was 10.67 (SD 6.33), for those with a current ED 16.61 (SD 5.84), and for those with no lifetime history of ED 6.83 (SD 5.58). Excellent internal consistency was demonstrated by Cronbach’s alpha’s for individuals with a previous ED (.88), for individuals with a current ED (.86), and for individuals with no history of ED (.88). Acceptable convergent validity was indicated based on significant correlations between the PHQ-9 and GAD-7 and DERS-SF. Confirmatory Factor Analyses revealed a mediocre fit for a one-factor structure of the PHQ-9, regardless of diagnostic status.

**Conclusions:**

The psychometric properties of the Norwegian version of the PHQ-9 are acceptable across females with and without ED, and the PHQ-9 can be recommended for use in clinical ED settings and for people without mental disorders.

**Supplementary Information:**

The online version contains supplementary material available at 10.1186/s12888-020-03013-0.

## Background

Depression is a common and serious mood disorder characterized by persistent feeling of sadness and hopelessness, loss of interest in previously enjoyed interests [1], and emotion regulation difficulties [2]. Commonly reported comorbid conditions include chronic somatic illness such as inflammatory bowel disease [3], diabetes [4], cardiovascular disease [5], and psoriasis [6], as well as psychiatric illness, including substance abuse [7], anxiety [8], and eating disorders (ED) [9]. ED are characterized by restricted or dysregulated food intake, distorted body image, and preoccupation with food, weight, and shape [1]. While general population prevalence estimates of depression vary from 17 to 31% [10], prevalence estimates of depression among individuals with ED have been reported to be as high as 75% [11]. However, estimates vary considerably depending on methodological approaches [12]. Depressive disorders are among the leading causes of worldwide burden, and are the second leading cause of years lived with disability [13]. Detection and treatment of depression is thus a public health priority.

Structured or semi-structured diagnostic interviews are designed to accurately determine psychiatric diagnoses, but require significant time and resources to conduct. In contrast, self-report assessment tools demand fewer resources to adopt and are easier to administer and score. Brief self-report questionnaires are an efficient way to screen individuals who score above a predetermined cut-off and may be in need of further clinical attention. Also, screening measures may be appropriate to use as an initial stage one in epidemiological studies prior to stage two diagnostic interviews. The high rates of chronicity and disability associated with depression [14] underscore the benefit of early screening and detection. A range of different self-report assessment tools have been used to measure symptoms and severity of depression, including Hospital Anxiety and Depression Scale (HAD) [15], the Beck Depression Inventory (BDI) [16], and the Patient Health Questionnaire-9 (PHQ-9) [17]. A recent systematic review investigated specificity and sensitivity of instruments used to grade severity of depression, and found that out of twenty reviewed instruments, the PHQ-9 was one of only three measures fulfilling the minimum criteria for sensitivity and specificity, with a reported sensitivity of 88% and specificity of 78% for the cut-off score of ≥10 [18]. This cut-off was established by the developers using an independent structured mental health professional interview as the criterion standard [17].

The PHQ-9 consists of nine items that measure depression symptoms and severity [19, 20]. Mixed findings have been reported with regard to factor structure. Whereas some studies have supported the originally established one-factor solution [21], other studies suggest a two-factor solution, with one cognitive/affective- and one somatic factor [22]. A previous Norwegian study of adolescents [23] supported a one-factor structure in a confirmatory factor analysis (CFA), but this has not yet been confirmed among Norwegian adults. As the PHQ-9 is extensively used in both clinical and research settings for psychiatric assessment, proper validation of different versions is important to make sure that the same construct is measured. Despite its widespread use in both clinical and research settings in Norway, only one prior study has investigated the psychometric properties of the PHQ-9 [23]. This study adapted the PHQ-9 to adolescents by shortening the time reference from fourteen to seven days. This underscores the need to confirm the psychometric properties of the Norwegian version of the PHQ-9 among adults to allow for comparisons across international studies. A recent study using PHQ-9 among college women who screened positive for an ED reported moderate depression across different ethnic groups, indicating that comorbid ED and depression can present in various ethnic groups [24]. Including patient samples (e.g., ED) in this effort will aid in determining whether the psychometric properties of the PHQ-9 extend beyond healthy individuals. Considering the high comorbidity between ED and depression, validation of the Norwegian PHQ-9 is needed for both clinical ED samples and controls [9]. Also, many symptoms of depression overlap with those of ED (e.g. weight loss, appetite), and it is therefore important to specifically investigate psychometric properties of the PHQ-9 in currently ill ED samples. In addition to ED psychopathology, depression is associated with emotion regulation difficulties and anxiety [25–27]. In their systematic review, Sloan et al. [28] found evidence for emotion regulation as a transdiagnostic treatment construct across various psychopathologies, including anxiety, depression, and eating disorders. Specifically, Fowler et al. [29] reported good construct validity of the DERS based on moderate correlations with depression and anxiety.

We investigated the psychometric properties of the Norwegian version of the PHQ-9 in adults with and without a lifetime ED diagnosis. Specifically, we investigated the internal consistency and convergent validity, attempted to confirm a one-factor structure, and present normative data. Convergent validity was explored by examining correlations with other theoretically related constructs, e.g. anxiety, ED psychopathology, and emotion regulation. We hypothesized that the Norwegian PHQ-9 would exhibit acceptable psychometric properties across ED diagnostic status.

## Methods

### Design and procedure

This cross-sectional case-control study is part of the Eating Disorders: Genes & Environment (EDGE) project, which investigates genetic and environmental risk factors for the development of ED. All Norwegian residents over the age of 16 years were eligible for participation. Individuals with a lifetime history (current or past) of an ED were invited to participate, as well as individuals without a lifetime history of an ED. Thus, a deliberate effort was made to recruit a diverse sample consisting of individuals with and without ED. There were no additional inclusion/exclusion criteria for our group of individuals with no history of ED. Therefore, controls may have mental health issues not directly assessed in our study. This strategy was intended; we did not want a “super healthy” control group; which runs the risk of maximizing between-group differences and reduce the validity of our findings. However, we note that the control group did score significantly lower across all psychopathologies assessed (ED, depression, anxiety). Participants were recruited through specialized ED treatment units across Norway, user-organizations for ED, online/social media platforms (e.g. websites, Facebook), and flyers and posters at Norwegian universities in the Oslo area. All participants completed an online assessment battery, collected between June 2019 and January 2020. The study was approved by the Regional Ethics Committee in Norway (project id: 2017/1606), and all participants provided informed consent.

### Participants

The sample consisted of 793 females aged 18–78 years, with a mean age of 30.39 years (SD 9.83). Mean BMI was 24.14 kg/m^2^ (SD 6.44). Based on self-report using the ED100K (see description below), a total of 19.7% of the participants had a previous history of the DSM-5 (1) ED diagnoses anorexia nervosa (AN), bulimia nervosa (BN), or binge eating disorder (BED), 36.3% of the participants were classified as having a current ED, and 44% of the participants had no history of ED. Among individuals with a current ED, 31.3% (*n* = 85) were classified as having AN, and 35.7% (*n* = 97) were classified as having BN/BED. BN and BED are combined due to difficulties of separating the two diagnoses, mainly due to diagnostic lifetime cross-over. Among individuals with a previous ED, a total of 43.9% (*n* = 65) of the participants had AN, and 35.8% (*n* = 53) had BN/BED. Participants were classified as “current ED” if they a) have a lifetime history of DSM-5 AN, BN, or BED on the ED100K; *and* b) have current ED symptoms (AN: BMI < 18.5 or frequent fasting; BN: episodes of binge eating and compensatory behaviors; BED: binge-eating episodes) on the ED100K; *and* c) score above the Norwegian EDE-Q cut-off (2.5) [30]; or d) have a lifetime history of ED *and* report currently receiving treatment for an ED (see description of ED100K and EDE-Q below). Individuals with ED were grouped into two groups; one comprising those with a previous but not current ED, and the other comprising those with a current ED. Groups differed significantly with respect to age and BMI (*p* > .01), in addition to completed education (*Χ*^*2*^[4] = 14.97, *p* = .005) and employment status (*Χ*^*2*^[4] = 31.92, *p* < .001). Follow-up tests revealed that individuals with EDs were more likely to have lower education and to be unemployed (including on sick leave or welfare). These effects were driven by the individuals who were currently ill; while the recovered ED group had similar education and employment status to controls. In addition to these two groups of cases, a third control group consisted of individuals with no lifetime history of ED. Thus the sample was divided into three different groups depending on diagnostic status; current ED, previous ED and no ED. To ease readability, these three groups are sometimes referred to as individuals with and/or without ED. Participant characteristics for cases and controls are shown in Table 1.

**Table 1:** Participant characteristics

	Previous ED *N* = 148 (18.7%)	Current ED N = 272 (34.3%)	No ED history *N* = 330 (41.6%)
Age	31.78 (9.97)	29.44 (8.83)	30.72 (10.44)
BMI	23.61 (5.86)	24.26 (8.44)	24.02 (4.62)
Age of onset	15.18 (3.04)	15.11 (5.10)	NA
DERS-SF total score	49.63 (14.10)	59.06 (13.05)	39.70 (13.00)
GAD-7 total score	8.95 (5.37)	12.75 (5.04)	6.14 (4.63)
EDE-Q global score	1.88 (1.26)	4.13 (1.10)	1.29 (1.24)
EDE-Q restraint subscale	1.53 (1.46)	3.74 (1.57)	1.08 (1.32)
Binge eating episodes last 28 days	1.22 (3.76)	9.66 (33.34)	.47 (2.61)
Self-induced vomiting episodes last 28 days	.36 (1.35)	13.65 (50.61)	.29 (3.66)
Laxative misuse episodes last 28 days	.22 (2.34)	1.78 (7.48)	.15 (1.79)
Education			
- *Primary/lower secondary school*	2.7%	8.5%	2.7%
- *Upper secondary school*	32.4%	38.2%	30.3%
- *University ≤ 4 years*	33.1%	33.1%	35.2%
- *University > 4 years*	28.4%	16.9%	30.0%
- *Other*	3.4%	3.3	1.8%
Work			
- *Studies*	34.5%	30.1%	42.7%
- *Full time employed*	35.1%	24.3%	34.2%
- *Part-time employed*	12.2%	10.3%	9.4%
- *Other employment*	3.4%	5.9%	4.8%
- *Unemployed, welfare, sick leave, temporarily laid off*	14.9%	29.4%	8.8%

*Note*: Data on age, BMI, and DERS total score are reported as means (SD), education and work as percentages. *ED* eating disorders; *BMI* body mass index; *DERS-SF* Difficulties in Emotion Regulation Scale Short Form; *GAD* Generalized Anxiety Disorder; *EDE-Q* Eating Disorder Examination Questionnaire; *NA* Not Applicable

### Measures

A Norwegian translation and adaptation of the ED100K self-report measure [31] was utilized to assess lifetime history of AN, BN, and BED according to DSM-5 criteria. This measure contains approximately 84 items probing lifetime frequency, duration, and severity of core ED symptoms, including binge-eating, compensatory behaviors, and weight history, as well as age when these features first emerged (i.e. age of onset). Responses are recorded using several formats, designed to clearly capture ED features fulfilling DSM-5 criteria. Due to the retrospective design of this study, and the significant cross-over between the different ED types, a considerable proportion of our sample had a history of several ED diagnoses and subtypes (e.g. AN and BN). Because of this, we did not perform additional analyses according to ED diagnoses or subtypes, as the resulting sample sizes would be too small for our psychometric investigation. The ED100K has previously been validated against the Structured Clinical Interview for DSM-5 (SCID), which has documented good predictive validity [31]. In our study, a total of 74% of individuals classified as having a current ED reported to have received ED treatment. For individuals classified with a previous ED or with no lifetime ED, the number of participants reporting to have received ED treatment was 68.2 and 3.3% respectively. This further supports the validity of the ED100K. Self-reported data on weight and height were used to calculate body mass index (BMI).

The Patient Health Questionnaire-9 (PHQ-9) [17] consists of nine items and assesses depression symptoms the previous fourteen days. Responses are scored on a Likert scale ranging from 0 (*not at all*) to 3 (*nearly every day*). A predetermined cut-off of ≥10 is recommended for screening purposes. In addition to a total score, categories are defined to indicate severity of depression symptoms: *none* (total score ranging from 0 to 4), *mild* (total score ranging from 5 to 9), *moderate* (total score ranging from 10 to 14), *moderately severe* (total score ranging from 15 to 19), and *severe* (total score ranging from 20 to 27) [17]. The PHQ-9 was adequately translated and adapted to Norwegian through a translation-back-translation approach, in line with recommendations [19].

The Generalized Anxiety Disorder (GAD-7) scale [32], validated in Norwegian [33], was used to assess symptoms of anxiety. The GAD-7 is a self-report measure of anxiety consisting of seven items, and answers range from 0 (*not at all*) to 3 (*nearly every day*). Excellent internal consistency was found in the present study for participants with a previous ED (α = .90), current ED (α = .87), and no history of ED (α = .87).

Eating Disorder Examination – Questionnaire (EDE-Q) assessed ED psychopathology. The EDE-Q consists of the four subscales *eating restraint*, *eating concern*, *shape concern*, and *weight concern*. As the literature provides mixed support for these subscales [34–36], the present study reports the overall global score only [34]. Answers are ranged from 0 (least frequent/severe) to 6 (most frequent/severe). Excellent internal consistency was found in the present study for participants with a previous ED (α = .93), current ED (α = .90), and no history of ED (α = .95).

The Difficulties in Emotion Regulation Scale Short Form (DERS-SF) [37] is a widely used self-report measure of emotion regulation deficits. The DERS-SF consists of 13 items, which is summed to produce a total score. Answers range from 1 (almost never) to 5 (almost always). Excellent internal consistency was found in the present study for participants with a previous ED (α = .85), current ED (α = .85), and no history of ED (α = .86).

### Statistical analyses

Pearson correlation analyses were carried out to investigate convergent validity; i.e., to assess whether the PHQ-9 total score correlated with other constructs hypothesized to be associated with depression, such as symptoms of anxiety (GAD-7 score) and ED (EDE-Q global score). In line with Cohen [38], correlations of .10 to .29 were interpreted as small, .30 to .49 as medium and .50 to 1.0 as large. Furthermore, due to violating the ANOVA assumptions of equal variance, a non-parametric Kruskal-Wallis H test was conducted to compare PHQ-9 scores across levels of ED groups (current/previous ED cases versus comparisons). Interquartile range (IQR) was calculated for the three groups. Mann-Whitney U tests were performed for post-hoc analyses, with alpha level .017 subsequent to Bonferroni correction (.05/3) for multiple comparisons. Effect sizes (r) were calculated and classified using Cohen’s classification, with .01 as small effect, .06 as a medium effect, and .14 as a large effect. Cronbach’s alpha was calculated to indicate internal consistency for the PHQ-9 scale, and confirmatory factor analysis (CFA) was used to seek confirmation of the original one-factor solution as reported by Kroenke et al. [17], and confirmed among Norwegian adolescents [23].

We used maximum likelihood estimation in the CFA. The current analytic approach was undertaken in two phases. First, a CFA-model was fitted to the data. The second phase involved the use of multiple indicators, multiple causes (MIMIC) modeling to investigate whether the latent factor mediates the effect of the observed severity group on the latent construct of the PHQ-9 in those with and without eating disorder and to investigate differential item functioning (DIF). DIF occurs when an item on a test or questionnaire has different measurement properties for one group of people versus another, irrespective of group-mean differences on the variable under study. We tested for differential item functioning, by comparing the fit of the model using log-likelihood tests, in different conditions [39].

In a MIMIC-approach, a direct path to the latent construct indicates the effects of the group contrast. Following a similar procedure as in previous work [33], multiple indices were used to evaluate the models. Different indices provide different information (i.e., absolute fit, fit adjusting for model parsimony, fit relative to a null model), and more indices give a more conservative and reliable evaluation of the model fit [40]. The chi-square distribution for goodness of fit evaluates the difference between the observed data and model prediction. For the comparative fit index [CFI [41];] and the Tucker-Lewis Index [TLI [42];], a value of 0.95 suggests acceptable fit. For root mean square error of approximation [RMSEA [43];], values in the range of 0.00 to 0.05 indicate close fit, those between 0.05 and 0.08 indicate fair fit, and those between 0.08 and 0.10 indicate mediocre fit. RMSEA values above 0.10 indicate poor fit. Standardized root mean square residual (SRMR) is an absolute measure of fit and is defined as the standardized difference between the observed correlation and the predicted correlation. A value below 0.08 is generally considered a good fit [41].

The CFA was conducted using mplus version 8, whereas IBM SPSS statistics version 25 was used for the remaining analyses.

## Results

### Normative data

Mean PHQ-9 total scores were calculated for the three diagnostic groups, resulting in a mean score of 10.67 (SD 6.33) for those with a previous history of ED, 16.61 (SD 5.84) for those with a current ED, and 6.83 (SD 5.58) for those with no lifetime history of ED. Proportion of participants falling within the different severity categories (none, mild, moderate, moderately severe, and severe) are illustrated in Table 2. Briefly, 12.7% of participants with no lifetime history of ED fell within the two most severe categories (total PHQ-9 score of 15 or above; moderately severe and severe depression symptoms). For participants with a previous or current ED, these rates were 29 and 63.9%, respectively. A total of 52.8% of the total sample scored above the PHQ-9 cut-off score of ≥10. When analyzing separately according to diagnostic status, 53.4% of individuals with a previous ED scored above the cut-off, 86.4% among individuals with a current ED, and 26.1% of participants with no lifetime history of ED scored above the cut-off score. Item-level mean scores are presented in Table 3.

**Table 2:** Proportion of individuals falling within the different PHQ-9 severity categories

PHQ-9 severity range	Previous ED N = 148 (18.7%)	Current ED N = 272 (34.3%)	No lifetime ED N = 330 (41.6%)
None (score 0–4)	*N* = 30 (20.3%)	N = 7 (2.6%)	*N* = 143 (43.3%)
Mild (score 5–9)	*N* = 39 (26.4%)	N = 30 (11.0%)	*N* = 101 (30.6%)
Moderate (score 10–14)	*N* = 36 (24.3%)	*N* = 61 (22.4%)	*N* = 44 (13.3%)
Moderately severe (score 15–19)	N = 27 (18.2%)	*N* = 82 (30.1%)	*N* = 31 (9.4%)
Severe (score 20–27)	*N* = 16 (10.8%)	*N* = 92 (33.8%)	*N* = 11 (3.3%)

*Note*: data are % of participants in the different ED diagnostic categories falling within each severity category. *ED* eating disorders; *PHQ* Patient Health Questionnaire

**Table 3:** Item-level scores of the PHQ-9

a)				
PHQ-9 item		Previous ED N = 148 (18.7%)	Current ED *N* = 272 (34.3%)	No ED N = 330 (41.6%)
1	Little interest/pleasure of doing things	1.13 (.95)	1.76 (.93)	.72 (.85)
2	Feeling down, depressed, or hopeless	1.12 (1.00)	1.83 (.98)	.70 (.84)
3	Trouble falling or staying asleep, or sleeping too much	1.55 (1.06)	2.07 (1.00)	1.14 (1.02)
4	Feeling tired or having little energy	1.74 (.99)	2.26 (.87)	1.36 (.96)
5	Poor appetite or overeating	1.28 (1.02)	2.26 (.90)	.89 (.96)
6	Feeling bad about yourself – or that you are a failure or have let yourself or your family down	1.53 (1.08)	2.43 (.79)	.90 (.96)
7	Trouble concentrating on things, such as reading the newspaper or watching television	1.17 (1.04)	1.87 (1.01)	.68 (.87)
8	Moving or speaking so slowly that other people could have noticed, or so fidgety or restless that you have been moving a lot more than usual	.57 (.83)	.90 (.93)	.22 (.56)
9	Thoughts that you would be better off dead, or thoughts of hurting yourself in some way	.58 (.90)	1.21 (1.12)	.22 (.60)
**b)**				
**Items**	**Factor loading**			
Little interest/pleasure of doing things	0.847			
Feeling down, depressed, or hopeless	0.861			
Trouble falling or staying asleep, or sleeping too much	0.711			
Feeling tired or having little energy	0.773			
Poor appetite or overeating	0.784			
Feeling bad about yourself – or that you are a failure or have let yourself or your family down	0.846			
Trouble concentrating on things, such as reading the newspaper or watching television	0.806			
Moving or speaking so slowly that other people could have noticed, or so fidgety or restless that you have been moving a lot more than usual	0.624			
Thoughts that you would be better off dead, or thoughts of hurting yourself in some way	0.756			

*Note*: Data are mean scores and standard deviations. *ED* eating disorders; *PHQ* Patient Health Questionnaire

*Note*: Standardized factor loadings from the modified confirmatory one-factor factor analysis (CFA)

A Kruskal-Wallis H test revealed a statistically significant difference in PHQ-9 scores between the groups, *Χ*^2^(2) = 274.27, *p* < .001 (Table 4), with a mean rank PHQ-9 score of 244.8 for individuals with no lifetime history of ED (IQR = 3–10), 368.0 for individuals with previous ED (IQR = 5–15), and 538.21 for individuals with a current ED (IQR = 12–21). Mann-Whitney U tests were performed for post-hoc analyses, and statistically significant differences in PHQ-9 total scores were revealed in all pairwise comparisons (see Table 4 for details).

**Table 4:** PHQ-9 scores across ED group status in a Kruskal-Wallis test with Mann-Whitney U post hoc pairwise tests

***Non-parametric K independent ANOVA***							
**Group**	**N**	**Md**	**IQR**	**Mean ranks**	**Kruskal-Wallis H**	**df**	**Significance level**
Previous ED	148 (18.7%)	10	5–15	367.96	274.27	2	.001
Current ED	272 (34.3%)	17	12–21	538.21			
No lifetime ED	330 (41.6%)	5	3–10	244.77			
***Mann-Whitney U tests (*post hoc** ***pairwise comparisons)***							
**Comparison**	**U**	**z**	**Significance level**	**Effect size (r)**			
Previous ED vs current ED	9997.00	−8.53	.001	−.42			
Previous ED vs no lifetime ED	15,405.50	−6.47	.001	−.30			
Current ED vs no lifetime ED	10,753.00	−16.08	.001	−.66			

*Note*: alpha level set at .017 after Bonferroni correction for multiple comparisons. *ED* eating disorders; *PHQ* Patient Health Questionnaire; *ANOVA* analysis of variance; *Md* median; *IQR* interquartile range; *df* degrees of freedom

### Internal consistency of the PHQ-9

Excellent internal consistency was indicated by Cronbach’s alphas for the total sample (.92), for individuals with a previous ED (.88), for individuals with a current ED (.86), and for individuals with no history of ED (.88).

### Convergent validity

Convergent validity was indicated by positive significant correlations with the GAD-7, EDE-Q, and DERS-SF total scores; constructs expected to be associated with depression. Correlation analyses showed that the PHQ-9 and GAD-7 were significantly associated in the total sample (.81, *p* < .001). Moreover, the PHQ-9 was correlated with the EDE-Q global score (.71, *p* < .001) and the DERS-SF total score (.76, *p* < .001). When separated by diagnostic group, the PHQ-9 score was associated with GAD-7 (.79, *p* < .001), EDE-Q (.38, *p* < .001), and DERS-SF (.63, *p* < .001) among participants with a previous ED. For participants with a current ED, PHQ-9 was associated with GAD-7 (.64), EDE-Q (.61), and DERS-SF (.64), all *p*’s < .001. Finally, the corresponding correlations for individuals with no lifetime history of ED were .81 (*p* < .001), .53 (*p* < .001), and .70 (*p* < .001). Correlations between these constructs, in addition to age, and BMI, are illustrated in Table 5, separated by groups.

**Table 5:** PHQ-9 correlations with anxiety, ED psychopathology, emotion regulation difficulties, age, and BMI

	Previous ED N = 148 (18.7%)	Current ED N = 272 (34.3%)	No history of ED N = 330 (41.6%)
GAD scale total score	.79***	.64***	.81***
EDE-Q global score	.38***	.61***	.53***
DERS total score	.63***	.64***	.70***
Age	ns	ns	−.11*
BMI	ns	−.20**	.11*

*Note*: *** indicates statistical significance = *p* < .001, ** = *p* < .01, and * = *p* < .05. *ED* eating disorders; *PHQ* Patient Health Questionnaire; *GAD* generalized anxiety disorder; *EDE-Q* Eating Disorder Examination – Questionnaire; *DERS* Difficulties in Emotion Regulation Scale; *BMI* body mass index

### Factor structure

**Fig. 1:**
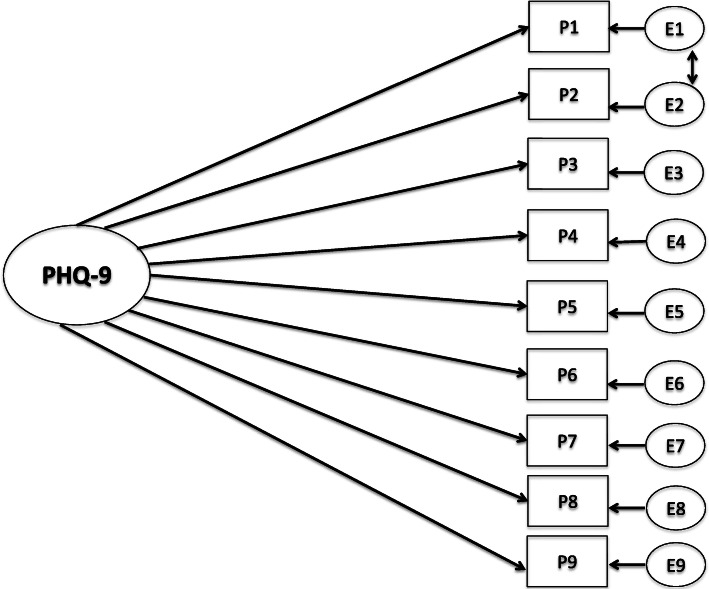
Confirmatory factor analysis of the PHQ-9. P1 = Lack of interest, P2 = Depressed, P3 = Sleep, P4 = Energy, P5 = Appetite, P6 = Feeling bad, P7 = Concentrating, P8 = Speaking slowly/restless, P9 = Suicidal thoughts. E = residuals

The one-factor model of PHQ-9 provided a poor fit for the sample (χ^2^_28_[*N* = 762]. 302.85; *p* = 0.00; CFI = .93; TLI = .91; RMSEA = 0.113; [0.102–0.125]; SRMR = 0.089. Thus, the one-factor solution was not confirmed. However, when evaluating the modification indices, evidence of correlated residuals for items 1 and 2 where found, similar to other studies [44]. Thus, the CFA was specified again by freely estimating the error covariances of these item one and item two (see Fig. 1). The revised model gave a better model fit; however, the fit indices were still mediocre (χ^2^_27_[*N* = 762]. 211.188; *p* = 0.00; CFI = .95; TLI = .94; RMSEA = 0.095; [0.08–0.11]; SRMR = 0.107. Since the fit was mediocre, a two-factor solution proposed in the literature with cognitive and somatic symptoms was investigated [45]. The two-factor solutions gave somewhat better model-fit (χ^2^_27_[*N* = 762]. 170.654; *p* = 0.00; CFI = .96; TLI = .95; RMSEA = 0.084; [0.07–0.10]; SRMR = 0.130), but the factors were highly correlated (*r* = 0.93) as previously reported [44], thus we proceeded with the one-factor solution.

The response indicators defining the one-factor model reported in Fig. 1 were inserted in the entire MIMIC model. The contrast variable represented those with and without ED and was regressed on the latent construct (PHQ-9). The MIMIC model indicated a worse fit than the respecified one-factor CFA-model: (χ^2^_35_[N = 762]. 316.219; *p* = 0.00; CFI = .93; TLI = .92; RMSEA = 0.103; [0.09–0.11]; SRMR = 0.150). Thus, including a covariate in the measurement model, indicating ED or not, did not improve the model fit. Our MIMIC model confirms that patients without ED were more likely to have lower scores on the PHQ-9 (− 1.02, *p =* < 0.001) than patients with a history of or current ED. Every item was tested for uniform DIF with all other items presumed DIF-free. This was accomplished by regressing one item at a time on the grouping variable. A full model (every items DIF-free) was compared to a more constrained model using log-likelihood tests, where one of the items were regressed to the grouping variable. The results indicated that the items were invariant in those with and without ED.

## Discussion

The overarching aim of this study was to investigate the psychometric properties of the Norwegian version of the PHQ-9 in a female adult sample with and without a lifetime history of ED. The results suggest that the psychometric properties are generally good, with excellent internal consistency and good convergent validity across diagnostic status. CFA revealed that a one-factor model of the PHQ-9 was the solution with the best fit to the data, though the fit was mediocre. No evidence of DIF was found based between those with and without ED. The results indicate that level of depression measured with PHQ-9 can be compared between such groups.

### Psychometric properties

The internal consistency of the Norwegian version of the PHQ-9 was excellent among adult females, with Cronbach’s alphas between .86–.92 for the different ED groups. This is similar to results from the previous Norwegian adolescent study (Cronbach’s alpha of .86 for the total sample and .88 for girls only) [23], as well as other studies among adult (male and female) samples [17, 45], all reporting Cronbach’s alphas between .79 and .89. Thus, reported internal consistency was similar across gender in these studies. However, it should be noted that this does not necessarily mean that the results in the current study can be generalized to males. Furthermore, the PHQ mean score in our study was positively and strongly associated with ED psychopathology, emotion regulation difficulties, and anxiety. These are constructs theoretically related to depression, thereby indicating convergent validity [25, 26, 28]. While scores on PHQ-9 showed moderate to large correlations with scores on ED psychopathology and emotion regulation, associations between scores on depression and anxiety were large. Although the meaningfulness of separating the constructs of anxiety and depression can be debated, here it supports satisfactory convergent validity of the Norwegian translation of the PHQ-9.

A one-factor model of the PHQ-9 was the solution with the best fit to the data, even though the fit was mediocre. The one-factor model exhibit strong factor loading (0.63–0.86) and high internal consistency (.86–89). This contrasts with some aspects of the existing literature [21], including a Norwegian study of adolescents [23], yet other studies have reported a two-factor structure [22], including a somatic and a cognitive/affective factor. These contradictory factor structure findings may reflect sample differences, although analyses of measurement invariance indicate that PHQ-9 is a reliable and valid measure across demographic groups [21, 44]. Furthermore, it has been argued that since the factors in the two-factor structure are highly correlated (.86), it is of limited value to distinguish them [44]. Additionally, the PHQ-9 is a brief, nine-item measure designed to effectively screen for depression, which could suggest that using the total score of the PHQ-9 to indicate depression severity may be beneficial when using the measure clinically and in research.

### Normative data

Normative data were also presented, demonstrating higher PHQ-9 mean scores in the ED groups compared to those with no lifetime ED. Though elevated scores may be expected among individuals with a previous ED, it is worth noting that the PHQ-9 mean score of the individuals with no ED history in our study is considerably higher than that reported among representative nationwide population-based samples from other studies, such as Germany [46], the USA [22], and South Korea [45]. Mean scores for males and females in these studies typically range from 2.5 to 4.5, though females tend to score somewhat higher than males. There were no exclusion criteria for our group of individuals with *no history of ED* with the exception of lifetime history of ED. It is therefore possible that individuals in this group have other mental health problems. Supporting this, 18.6% of the individuals in the comparison group reported currently receiving mental health treatment, although this does not necessarily signal the presence of a psychiatric disorder. This could indicate that our sample of people with no ED history is not as healthy as those in larger population studies that strive for super healthy controls. Notably, the Norwegian adolescent study reported similar norms to the current Norwegian adult study, with a mean score of 6.89 (SD 5.13) in adolescent females [23]. It can therefore not be ruled out that differences in normative data across studies reflect cultural differences in symptomatology or reporting; however, this cannot be concluded based on our data.

Other studies of clinical samples of individuals with ED have reported PHQ-9 mean scores falling in the same range as the past- and current ED groups in the present study [47, 48]. For example, Hayes et al. [47] reported normative data in study of adolescents and adults (93% females) with ED receiving treatment with a partial hospitalization and intensive outpatient program. Baseline PHQ-9 mean score was 12.79 (SD 6.91), dropping to 8.12 (SD 6.91) post treatment. Furthermore, Rose et al. [48] reported PHQ-9 mean scores in adults (44 females and 3 males) with ED in primary care pre and post CBT for ED (mean number of sessions 17). Baseline mean was 13.5 (SD 5.48), post treatment was 7.42 (SD 6.38). Based on these studies, it may seem like baseline PHQ-9 mean scores of ED clinical samples resemble those of the two clinical groups in the present study (mean scores 10 in the previous ED group and 16 in the current ED group), whereas post-treatment scores in the clinical studies fall closer to the group with no lifetime ED in the present study. These studies consisted of predominantly female samples, but Hayes et al. reported that gender was not a significantly moderator for any of the outcome measures.

Furthermore, with regard to prevalence, a total of 53.4% with a previous ED, and 86.4% of individuals with a current ED, scored above the PHQ-9 depression screening cut-off score of 10 in the present study. As expected, the proportion of individuals with no lifetime ED history scoring above cut-off, was considerably lower at 26.1%. However, these scores are still noticeably higher than the German population data [46], reporting that 5.6% of all participants scored above the cut-off of 10. As noted above, it cannot be determined whether these differences relate to cultural-, selection-, or other factors. Whereas the national German population study had a representative registered-based sample, our study mainly utilized online recruitment. Such various recruitment approaches may affect the samples attained, thereby potentially bias the results. It is a possible that cut-off thresholds may need to be culturally adapted. To achieve this, two-stage studies are needed.

Our findings suggest the Norwegian version of the PHQ-9 is a reliable and valid measure that can be used to assess depression symptoms among female individuals with ED. This is important as depression symptoms often co-occur with ED, and monitoring such symptoms may be of importance to assess treatment response. Moreover, our normative data showed that depression scores were elevated among recovered individuals who have a history of an ED. This has implications for the interpretation of PHQ-9 scores among such individuals. Because symptoms of depression overlap with those of ED (e.g. weight loss, appetite), future studies should evaluate whether the traditional PHQ-9 cut-off thresholds are equally valid for ED populations.

Although this study is strengthened by a large sample with and without ED, it is limited by the use of self-report data to ascertain lifetime ED diagnoses. Also, we cannot rule out that our online recruitment procedure may have affected the results. Differences across the different ED diagnoses were not addressed. Though the psychometric properties of the Norwegian translation of the PHQ-9 are found to be good among females, diagnostic interviews are required to determine diagnoses. Also, males are not included in the study. This limits the generalizability across genders and confidence in gender-specific norms. Finally, another measure of depression was not included, which would have strengthened evidence in favor of the construct validity of the PHQ-9.

## Conclusions

The results indicate that the Norwegian version of the PHQ-9 is psychometrically sound among females across different ED diagnostic group status and females with no lifetime ED. This suggests that PHQ-9 can be used in ED clinical settings as well as in the general Norwegian population to assess depression symptoms and severity [14].

## Supplementary Information

**Additional file 1.**

## Data Availability

The datasets used and/or analyzed during the current study are available from the corresponding author on reasonable request.

## Abbreviations

PHQ: Patient health questionnaire
EDE-Q: Eating disorder examination questionnaire
GAD: Generalized anxiety disorder
DERS-SF: Difficulties in emotion regulation scale short form
ED: Eating disorders
CFA: Confirmatory factor analysis
EDGE: Eating disorders genetic and environment
DSM: Diagnostic and statistical manual for mental disorders
AN: Anorexia nervosa
BN: Bulimia nervosa
BED: Binge eating disorder
BMI: Body mass index
IQR: Interquartile range
ANOVA: Analyses of variance
SD: Standard deviation
