# Supplementary figures and images for: Psychometric properties of the Norwegian version of the Patient Health Questionnaire-9 (PHQ-9) in a large female sample of adults with and without eating disorders
Source: BMC Psychiatry. 2021 Jan 5;21:6. doi: 10.1186/s12888-020-03013-0 (PMC7786911; doi:10.1186/s12888-020-03013-0)

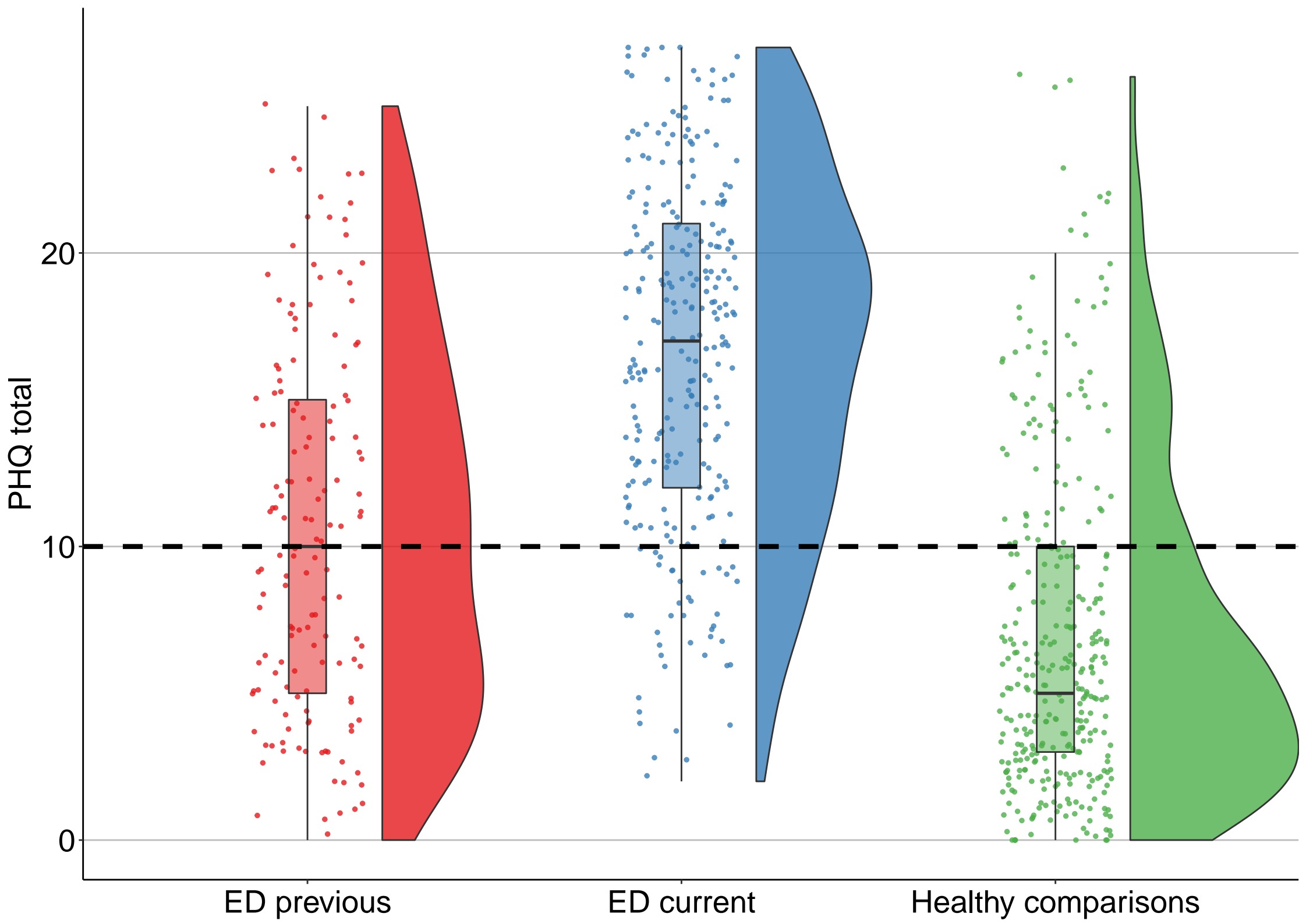

Supplement: Supplementary file 1 — Additional file 1. [file 12888_2020_3013_MOESM1_ESM.pdf]
